# Serum level and polymorphisms of retinol-binding protein-4 and risk for gestational diabetes mellitus: a meta-analysis

**DOI:** 10.1186/s12884-016-0838-7

**Published:** 2016-03-14

**Authors:** Shimin Hu, Qian Liu, Xin Huang, Hongzhuan Tan

**Affiliations:** 1 Department of Epidemiology and Health Statistics, School of Public Health of Central South University, Changsha, Hunan 410007 PR China; 2 Criminal Investigation Division, Changsha Public Security Bureau, Hunan, 410004 China

**Keywords:** Retinol-binding protein-4, Gestational diabetes mellitus, Meta-analysis

## Abstract

**Background:**

Retinol-binding protein-4 (RBP4) has been reported to be potentially involved in the pathogenesis of gestational diabetes mellitus (GDM); however, the findings are inconsistent. Our aims were to review the studies that investigated the association of serum levels and polymorphisms of RBP4 with GDM risk, and to provide recommendations for future research.

**Methods:**

The databases PubMed, EBSCO, ScienceDirect, and Web of Knowledge were searched up to October 2015 to find out studies evaluating the relationship between serum RBP4 level/ RBP4 polymorphisms and GDM risk. In the meta-analysis of serum RBP4 levels the key inclusion was that studies were designed as BMI-matched studies or had observed non-significant differences in BMI between cases and controls.

**Results:**

Fourteen case–control studies (647 cases and 620 controls) reporting the association between serum RBP4 level and GDM risk, and three studies (1012 cases and 1605 controls) investigating the association between *RBP4* polymorphisms and GDM risk were involved. Our results showed that high serum RBP4 levels represent a risk factor for GDM (pooled standardized mean difference =0.758, 95 % confidence interval [0.387, 1.128]). The results of subgroup analyses based on “gestational age at blood sampling” or “diagnostic criteria” are consistent with the overall results. However, the postpartum subgroup and “before 24 weeks” subgroup both only include one article and indicate no association between serum RBP4 level and GDM risk. The meta-analysis on the association between rs3758539 polymorphism and GDM risk shows that *RBP4* rs3758539 polymorphism is not associated with the development of GDM.

**Conclusions:**

The results of this meta-analysis support the hypothesis that RBP4 is a modest independent risk factor for GDM (i.e., nonobese patients with GDM might express RBP4 at abnormal levels). The serum RBP4 level is associated with the risk of GDM. However, the association in the first-trimester and postpartum period should be validated by further research. The association between RBP4 rs3758539 polymorphism and GDM risk was not confirmed.

**Electronic supplementary material:**

The online version of this article (doi:10.1186/s12884-016-0838-7) contains supplementary material, which is available to authorized users.

## Background

Gestational diabetes mellitus (GDM) is defined as varying degrees of glucose intolerance that is first detected during pregnancy [1]. The prevalence of GDM is increasing in decades and floating from 1.7 to 11.6 % among populations [2]. Although considerable research effort has been focused on GDM, the pathophysiology of the disease remains incompletely understood. During pregnancy, insulin resistance will be enhanced physiologically. Insulin resistance can be further strengthened by some factors such as obesity, leading to high risk of GDM [3–5].

Retinol-Binding Protein-4 (RBP4) is mainly synthesized by hepatocytes and adipose tissues. It was identified in 2005 as an adipocytokine with potential capability in reducing insulin sensitivity and enhancing hepatic gluconeogenesis [6]. A multi-center clinical study [7] revealed that serum RBP4 levels correlated with the magnitude of insulin resistance in subjects with obesity, impaired glucose tolerance, or type 2 diabetes and in nonobese, nondiabetic subjects with a strong family history of type 2 diabetes. In nonobese subjects, decreased expression of glucose transporter type 4 (GLUT-4) in adipocytes predicts increased serum RBP4 levels and insulin resistance. Adipose-specific deletion of GLUT-4 was reported to lead to secondary defects in insulin action in muscle and liver. The mechanism by which a decrease in adipocyte GLUT-4 results in an increase in RBP4 expression is unknown, but it might involve sensing of glucose by adipocytes. RBP4 is regulated by GLUT-4. The downstream of RBP4’s regulatory mechanism is that an increase in serum RBP4 levels can induce hepatic expression of phosphoenolpyruvate carboxykinase, a gluconeogenic enzyme, to increase gluconeogenesis, and impair insulin signaling in muscles through decreasing the expression of phosphoinositide-3 kinase [8].

In patients with a history of GDM, insulin resistance exists before pregnancy but worsens during gestation. Insulin secretion is insufficient to compensate for the insulin resistance and this leads to hyperglycemia, which can be detected through routine glucose screening during pregnancy. Thus, chronic insulin resistance is a central component of the pathophysiology of GDM [9]. Furthermore, the cellular GLUT-4 content also decreases in patients with GDM [10]. The aforementioned findings have predictably given rise to the hypothesis that RBP4 might contribute to insulin resistance in GDM, much as it does in type 2 diabetes.

To date, several genetic variants that affect *RBP4* expression levels (e.g., rs3758539 and rs12265684) have been investigated for their potential association with the risk of GDM, but the reported findings are inconsistent [11–13]. False-negative results, inadequate statistical power, small sample sizes, and ethnic differences may contribute to the lack of reproducibility in genetic-association studies [14, 15]. Among the *RBP4* genetic variants examined in the studies to date, only the rs3758539 variant was analyzed more than once, in the USA, China, and Mexico, and therefore we only analyzed the association between *RBP4* genetic variants (rs3758539) and GDM in the present study.

The association between serum RBP4 levels and GDM risk has also been investigated, and, the reported results are also inconsistent [12, 13, 16–33]. Statistical association does not necessarily mean causal association. We propose 3 hypotheses regarding the positive statistical association between RBP4 levels and GDM risk: (1) GDM pathophysiology is related to obesity but not RBP4 levels, and serum RBP4 levels are elevated because of the increased size of adipocytes [7]; (2) RBP4 is a pathophysiological link between obesity and insulin resistance; (3) RBP4 is a modest independent risk factor for GDM (i.e., nonobese patients with GDM might express RBP4 at abnormal levels). If we want to prove serum RBP4 level is associated with GDM risk, considerable attention must be paid to body mass index (BMI) matched strategy. The positive results of studies with BMI-matched strategy are more credible to convince us that RBP4 is a modest independent risk factor for GDM. In meta-analysis about serum RBP4 level, we only included those studies in which BMI was matched, or showed a similar distribution in case and control groups, and then analyzed whether RBP4 is an independent risk factor for GDM.

Our aims were to review previous studies that investigated the association of serum RBP4 levels and *RBP4* polymorphisms with GDM risk, and to provide recommendations for future research.

## Methods

### Literature search

First, the databases PubMed, EBSCO, ScienceDirect, and Web of Knowledge were searched up to October 2015 to find out studies evaluating the relationship between serum RBP4 level and GDM risk. The following keywords were used: (“gestational diabetes mellitus” OR “gestational diabetes” OR GDM) AND (“retinol-binding protein-4” OR “retinol binding protein-4” OR “retinol binding protein 4” OR “retinol-binding protein 4” OR RBP4 OR RBP-4). Next, we systematically searched the same databases for studies that examined RBP4 polymorphisms in patients with GDM. The following keywords were used in this step: (“gestational diabetes mellitus” OR “gestational diabetes” OR “GDM”) AND (“polymorphism” OR “variant”) AND (“retinol-binding protein-4” OR “retinol binding protein-4” OR “retinol binding protein 4” OR “retinol-binding protein 4” OR “RBP4” OR “RBP-4”); furthermore, the names of specific polymorphisms were combined with “gestational diabetes.” All reference lists of the resulting primary research reports and relevant reviews were manually searched to identify additional eligible studies.

### Eligible studies and data extraction

Eligible studies included in this meta-analysis: (1) investigated the relationship between serum RBP4 levels and GDM risk, or between GDM and at least 1 genetic variant of *RBP4*; (2) included a case group of patients with GDM and a control group; (3) diagnosed GDM according to the oral glucose-tolerance test (OGTT); (4) provided data with mean and standard deviation (SD) or median and interquartile range (in the case of the meta-analysis of serum RBP4 levels); (5) were not animal studies; (6) were designed as BMI-matched studies or had observed non-significant differences in BMI between cases and controls (in the case of the meta-analysis of serum RBP4 levels). Studies with overlapping data were excluded from our analysis. We identified only one study that required additional information: Ping et al. [12] had not reported the *P* value of the difference in BMI between cases and controls in their analysis of serum RBP4 levels. However, we obtained Ping’s PhD dissertation, written in Chinese, and extracted this information from it. Data were extracted independently by two reviewers in consultation with a third, from each study included. The information regarding the first author, year of publication, study population (country and ethnicity), number of patients and controls, and diagnostic criteria were extracted. For genetic-association studies, information was extracted on the frequency of genotypes, the Hardy-Weinberg equilibrium (HWE) status, and the genotyping method used. For studies investigated serum RBP4 levels, information was extracted on serum RBP4 measurements, the mean and SD of serum RBP4 levels, and the gestational age at the time of blood sampling. When the standard error of the mean (SEM) was reported, SD was calculated as $SD=SEM/\sqrt{n}$. If the case or control groups were further divided into subgroups, the data from the subgroups were merged as n = n_1_ + n_2_, $\overline{x}=\frac{n_1\overline{x_1}+{n}_2\overline{x_2}}{n_1+{n}_2}$ and $\mathrm{S}\mathrm{D}=\sqrt{\frac{\left({n}_1-1\right)S{D}_1^2+\left({n}_2-1\right)S{D}_2^2+\frac{n_1{n}_2}{n_1+{n}_2}\left({\overline{x}}_1^2+{\overline{x}}_2^2-2{\overline{x}}_1{\overline{x}}_2\right)}{n_1+{n}_2-1}}$ [34]. When a study provided medians and interquartile ranges (instead of means and SDs), without the minimum or maximum values, we treated the medians as means and calculated the SDs, as SD = interquartile range/1.35 [35]. If the study provided the minimum and maximum, we imputed the means and SDs as described by Hozo et al. [36]. When a study had tested the blood sample by two or more different methods, the result of the enzyme immunometric assay was chosen here because it is the most commonly used method in the studies included in our meta-analysis.

### Statistical methods

The association between serum RBP4 level and GDM risk was estimated by calculating the pooled standardized mean difference (SMD) and 95 % confidence interval (CI). To analyze the potential influences of gestational age at blood sampling and diagnostic criteria, we performed subgroup analysis. Based on the gestational age at blood sampling, the subjects were divided into 4 subgroups: (1) before 24 weeks, (2) 24–28 weeks, (3) after 28 weeks, and (4) postpartum; For two studies [26, 28], the gestational ages at blood sampling considerably overlapped within 24–28 weeks; thus, these two studies were assigned to the gestational age subgroup of 24–28 weeks. And according to the diagnostic criteria studies were divided into two subgroups: (1) based on the American Diabetes Association (ADA) criteria [37], and (2) based on the more rigorous criteria established by the National Diabetes Data Group (NDDG) [38], the World Health Organization (WHO) [39], and Sun et al. [20]. The association between genetic factors (e.g., genotypes and alleles) and GDM was examined by using the Chi-square test or the Fisher exact test. The association of the *RBP4* rs3758539 polymorphism with GDM risk was assessed, by calculating the pooled odds ratio (OR) and 95 % CI, according to general, dominant, and recessive genetic models, and an allelic model [40, 41]. The significance of the pooled OR and SMD was determined using Z test, and the level was set at *p* < 0.05.

Heterogeneity among studies was assessed using the *Q* test and the *I* ^2^ statistic [42, 43]. When significant heterogeneity was observed (*P* < 0.1 in the *Q* test, and *I* ^2^ > 50 %), a random effects model was used for pooling data from the primary studies; if the heterogeneity was not significant, a fixed effects model was used. The HWE compliance of the controls in each study was assessed using the Chi-square test. Sensitivity analysis was performed by sequentially excluding individual studies to assess the stability of the results. Funnel-plot asymmetry was assessed using Egger’s linear regression test, *P* < 0.05 representing significant publication bias. If asymmetry was observed, contour-enhanced meta-analysis funnel plots were used to distinguish publication bias from other causes of asymmetry [44, 45]. All analyses were performed using STATA 12.0 software (Stata Corporation, College Station, TX), and all *p* values were 2-tailed.

## Results

### Main characteristics of eligible studies

With respect to the association between serum RBP4 levels and GDM risk, an initial search identified 232 records of potentially relevant studies from the databases included. Of these, 202 records were excluded based on their title and/or abstract: these were repetitive publications, reviews, reports of animal studies, and/or reports of studies that investigated either outcomes irrelevant to this meta-analysis or adipokines other than RBP4. A further 16 full-text articles were excluded because: ① six papers contain data that overlapped with other articles, ② seven papers reported different BMI distributions between the case and control groups (*P* < 0.05), ③ and three papers with the plasma RBP4 concentrations or unavailable data. Final selected 14 case–control studies included 647 cases and 620 controls were included in our meta-analysis (Table 1, Additional file 1: Table S1); the process of study selection is shown in Additional file 2: Figure S1(a).

**Table 1:** Detailed characteristics of all eligible studies for the association with serum RBP4 levels and GDM

Study	Year	Country	No of case	No of control	Diagnose criteria^b^	Gestational age at blood sampling	Test	BMI^c^	RBP4(μg/ml)^d^		*P*-value
									Case	Control	
Chan a [16]	2007	China, Han	20	20	NDDG	24–28 weeks	ELISA	Matched	0.0424 ± 0.0138	0.0320 ± 0.0087	0.007
Chan b [16]	2007	China, Han	20	20	NDDG	immediately after delivery	ELISA	Matched	0.0301 ± 0.0110	0.0309 ± 0.0100	0.811
Kim [17]	2008	South Korea	10	9	ADA	24–28 weeks	ELISA	*P* > 0.05	39.1 ± 6.3	30.0 ± 10.0	0.026
Lewandowski^a^[19]	2008	Austria	15	35	ADA	28 weeks	Unclear	Matched	53.9 ± 18.7	34.4 ± 12.4	<0.001
Sun [20]	2009	China, Han	32	30	OGTT confirmed	24 h before delivery	ELISA	Matched	27.0 ± 1.2	19.4 ± 1.8	<0.001
Klein a [21]	2010	Austria, Caucasian	63	38	ADA	24–28 weeks	ELISA	Matched	18.0 ± 3.7	16.9 ± 5.1	0.213
Klein b [21]	2010	Austria, Caucasian	63	38	ADA	33 weeks	ELISA	Matched	20.3 ± 7.2	17.9 ± 6.1	0.089
Su [23]	2010	China, Han	63	58	ADA	24–28 weeks	ELISA	Matched	41.6 ± 12.2	34.5 ± 9.8	<0.001
Tepper [24]	2010	USA, mixed	12	10	ADA	24–28 weeks	EIA	Matched	25.2 ± 2.1	25.2 ± 2.1	1.000
Kuzmicki a^a^[26]	2011	Poland	68	68	WHO	24–30 weeks	EIA	*P* > 0.05	58.1 ± 25.3	51.0 ± 18.1	0.062
Kuzmicki b^a^[26]	2011	Poland	20	18	WHO	36–40 weeks	EIA	*P* > 0.05	62.4 ± 31.0	42.9 ± 19.0	0.027
Chen [25]	2011	China, Han	52	46	NDDG	before delivery	ELISA	Matched	31.93 ± 7.21	26.92 ± 8.5	0.002
Ping [12]	2012	China, Han	74	69	ADA	24–28 weeks	ELISA	*P* > 0.05	21.53 ± 5.96	20.84 ± 4.31	<0.001
Skvarca^a^ [28]	2012	Slovenia	30	44	ADA	26.81 ± 3.46 weeks	ELISA	*P* > 0.05	15.00 ± 6.11	15.57 ± 8.35	0.750
Liang [31]	2014	China, Han	35	35	NDDG	24–28 weeks	ELISA	*P* > 0.05	22.90 ± 3.09	17.90 ± 3.91	<0.001
Fruscalzo [32]	2015	Germany, mixed	32	44	ADA	11–13 weeks	ELISA	*P* > 0.05	24.78 ± 6.51	27.93 ± 7.98	0.071
Du [33]	2015	China	38	38	NDDG	37–42 weeks	ELISA	Matched	39.08 ± 8.29	21.42 ± 3.85	<0.001

^a^Raw data in the article are presented as median (interquartile range)

^b^Diagnostic criteria: *NDDG* National Diabetes Data Group criteria, *ADA*, American Diabetes Association criteria, *WHO* World Health Organization criteria

^c^BMI: matched, the control subjects had been matched with the GDM subjects for BMI; *P* > 0.05, difference of BMI between case and control had no statistical significance

^d^Data are presented as mean ± standard deviation

With respect to the association between *RBP4* rs3758539 polymorphism and GDM risk, the initial database search identified 147 reports of potentially relevant studies. Of these, 143 records were excluded based on their title and/or abstract: these were repetitive publications, reviews, reports of animal studies, and/or reports of studies that investigated either outcomes irrelevant to this meta-analysis or genes other than *RBP4*. In addition, 1 full-text review was excluded because it contained data overlapped with other primary papers. Finally, 3 eligible studies that included 1,012 cases and 1,605 controls were included in the meta-analysis (Table 2); the process of study selection is shown in Additional file 2: Figure S1(b).

**Table 2:** Detailed characteristics of all eligible studies for the association with RBP4 gene polymorphisms and GDM

Gene polymorphisms	Author	Year	Country	Ethnicity	Case include	No of case	No of control	Genotype^a^		*P* for HWE
								Case	Control	
rs3758539	Hiraoka a^d^ [11]	2011	America	Utah Caucasian	GDM	88	315	56/31/1	228/77/10	0.27
	Hiraoka b^d^ [11]	2011	America	Hawai‘i Filipino	GDM	82	286	63/16/3	226/55/5	0.44
	Hiraoka c^d^ [11]	2011	America	Hawai‘i Pacific Islander	GDM	19	32	18/1/0	23/9/0	0.35
	Ping^e^ [12]	2012	China	Han	GDM + GIGT^c^	723	872	375/75/5	462/143/8	0.41
	Saucedo^d^ [13]	2014	Mexico	Unclear	GDM	100	100	93/7	91/9	*P* > 0.05^b^
rs12265684	Ping^e^ [12]	2012	China	Han	GDM + GIGT^c^	723	872	382/106/5	483/168/14	0.89
rs3758538	Ping^d^ [12]	2012	China	Han	GDM + GIGT^c^	723	872	414/63/4	562/76/2	0.74
rs10882273	Ping^d^ [12]	2012	China	Han	GDM + GIGT^c^	723	872	363/107/8	451/174/13	0.42
rs116736522	Saucedo^d^ [13]	2014	Mexico	Unclear	GDM	100	100	97/3	96/4	*P* > 0.05^b^
rs34571439	Saucedo^d^ [13]	2014	Mexico	Unclear	GDM	100	100	91/9	88/12	*P* > 0.05^b^

^a^Genotype for rs3758539, GG/AG/AA (For Saucedo, GG/GA + AA); rs12265684, CC/CG/GG; rs3758538, AA/AC/CC; rs10882273, TT/TC/CC; rs116736522, GG/GC + CC; rs34571439, AA/AC + CC

^b^No sufficient data to calculate the P for HWE. Authors mentioned “all variants are in the Hardy-Weinberg equilibrium” in the article

^c^ *GIGT* gestational impaired glucose tolerance

^d^No significant differences was found in allelic frequencies between case and control groups

^e^Significant differences was found in allelic frequencies between case and control groups

### Association between serum RBP4 level and GDM Risk

**Fig. 1:**
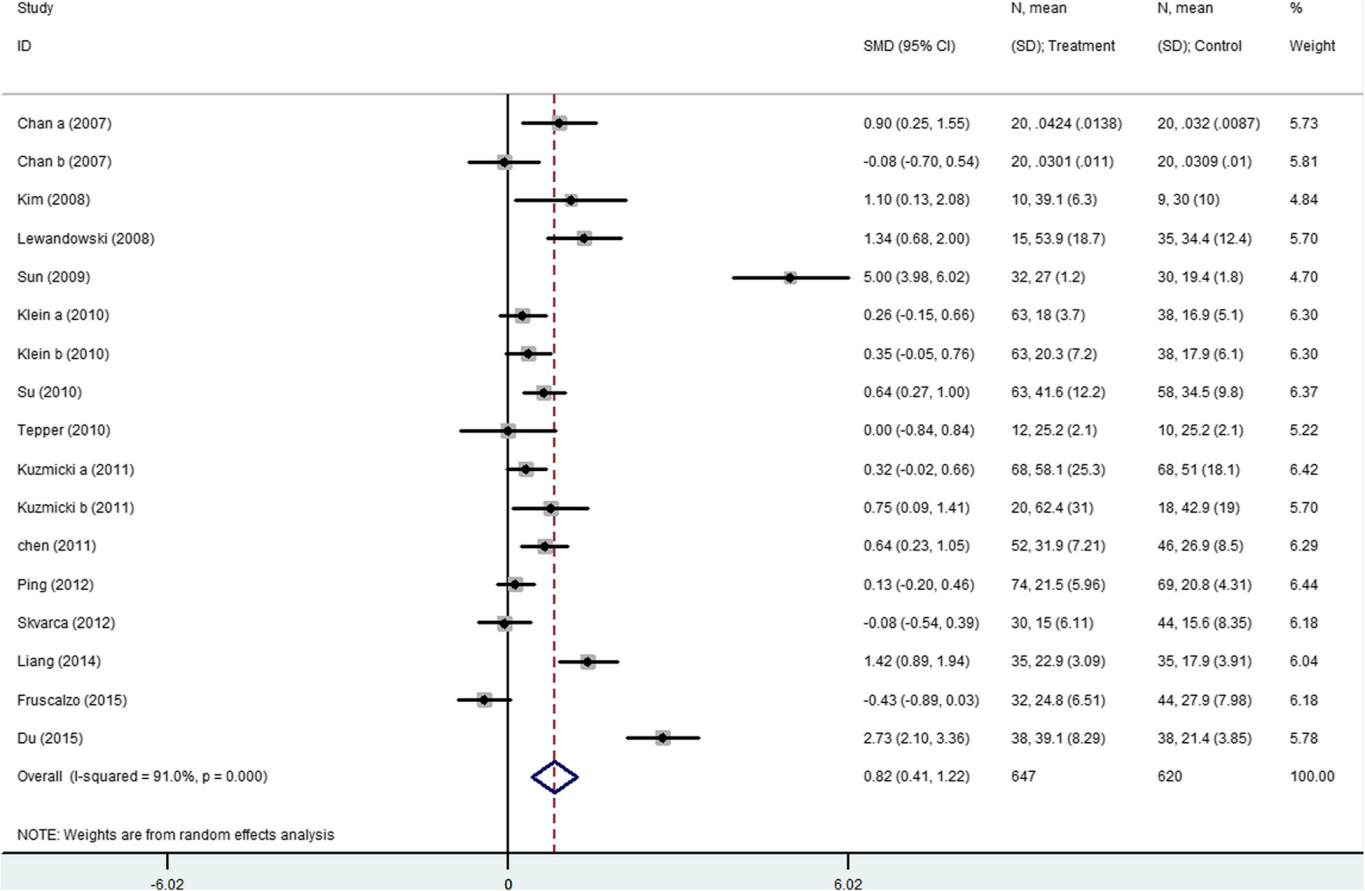
Meta-analysis for the association of serum RBP4 level with GDM risk using a random-effects model

**Fig. 2:**
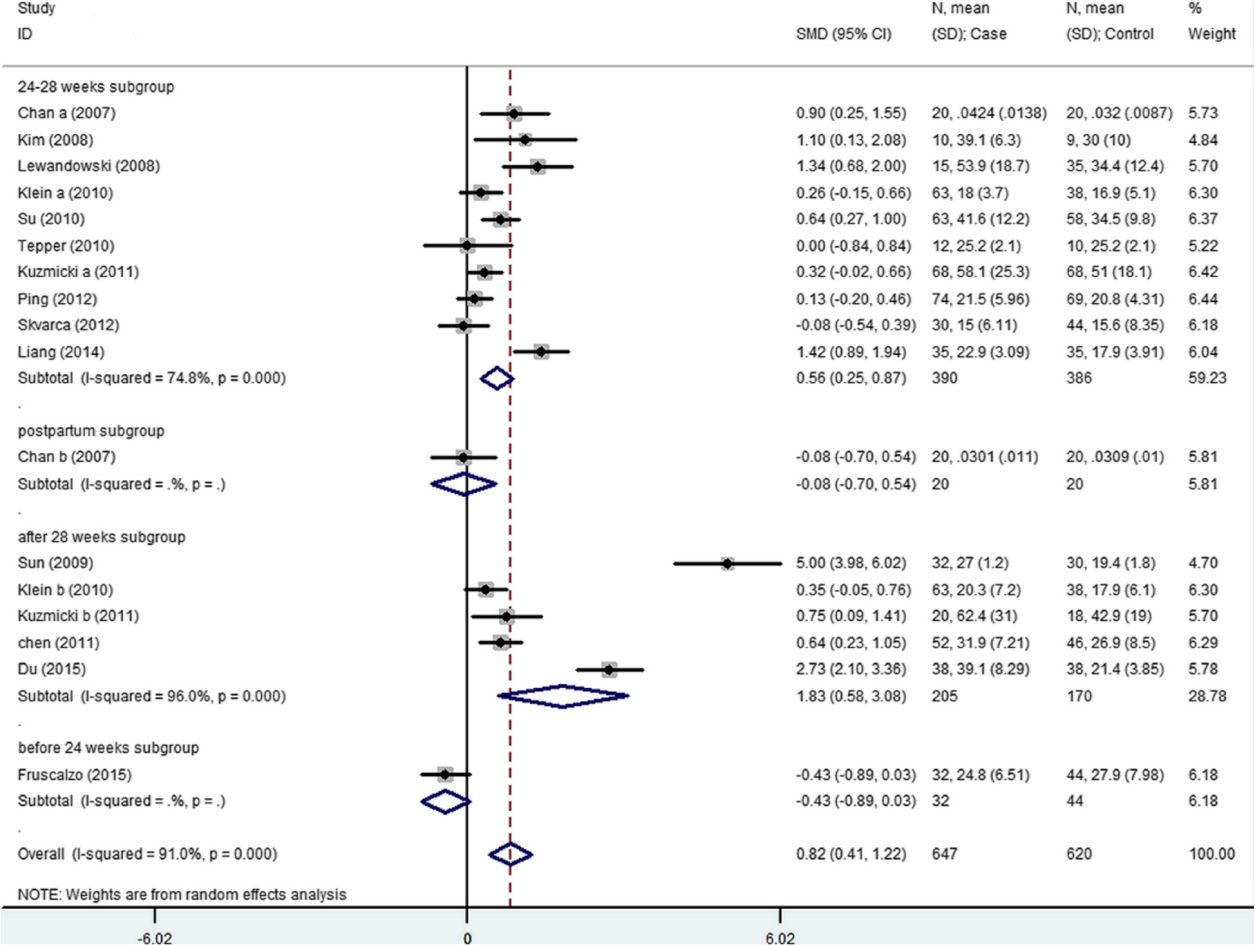
Gestational age at blood sampling subgroup analysis using a random-effects model

**Fig. 3:**
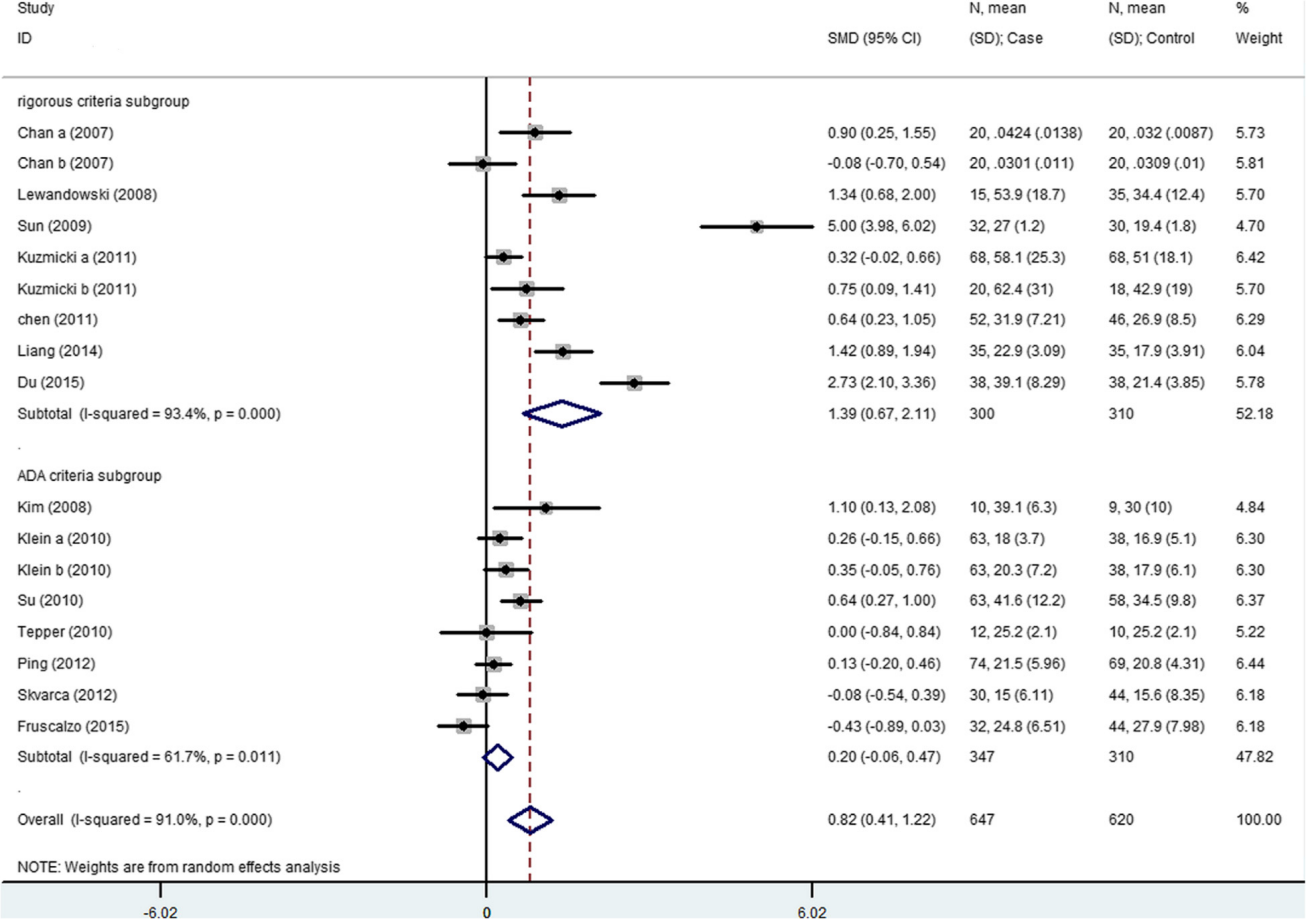
Diagnostic criteria subgroup analysis using a random-effects model

The meta-analysis for serum RBP4 level includes 14 articles (17 results) with a total of 647 cases and 620 controls. The results show that serum RBP4 level and GDM risk are significantly associated (SMD = 0.816, 95 % CI [0.411, 1.122]) (Table 3, Fig. 1). The “24–28 weeks” subgroup, “after 28 weeks” subgroup and the rigorous criteria subgroup indicate that higher serum RBP4 level is related to GDM risk (24–28 weeks subgroup: SMD = 0.561, 95 % CI [0.252, 0.870]; after 28 weeks: SMD = 1.830, 95 % CI [0.580–3.080]; the rigorous criteria subgroup: SMD = 1.388, 95 % CI [0.665, 2.110]). The “before 24 weeks” subgroup and postpartum subgroup both include only 1 study and show non-significant association between serum RBP4 level and GDM risk (before 24 weeks subgroup: SMD = -0.426, 95 % CI [-0.886, 0.870]; postpartum subgroup SMD = -0.076, 95 % CI [-0.696, 0.472]). Non-significant result is also observed in the ADA criteria subgroup (SMD = 0.205, 95 % CI [-0.063, 0.472]) (Table 3, Figs. 2 and 3).

**Table 3:** Summary of different comparative results of serum RBP4 level with GDM risk

Category		No of study	No of case	No of control	SMD (95 % CI)	Z	*p* value	I^2^%	*P*_het_
overall		17	647	620	0.816 [0.411–1.122]	3.94	0.000	91.0	0.000
Gestational age at blood sampling	Before 24 weeks	1	32	44	-0.426 [-0.886–0.035]	1.81	0.070	-	-
	24-28 weeks	10	390	386	0.561 [0.252–0.870]	3.56	0.000	74.8	0.000
	After 28 weeks	5	205	170	1.830 [0.580– 3.080]	2.87	0.004	96.0	0.000
	Postpartum	1	20	20	-0.076 [-0.696–0.544]	0.24	0.810	-	-
Diagnostic criteria	ADA criteria	8	347	310	0.205 [-0.063–0.472]	1.50	0.134	61.7	0.011
	Rigorous criteria	9	300	310	1.388 [0.665–2.110]	3.77	0.000	91.0	0.000

*P*_*het*_ = *p* value for heterogeneity, *OR* = adds ratio, *CI* = confidence interval, *ADA* = American Diabetes Association criteria, More rigorous criteria contained National Diabetes Data Group criteria, World Health Organization criteria and the criteria of Sun et al

In the sensitivity analysis, 1 eligible study was excluded at a time in order to assess the influence of each dataset on the pooled SMD. We observed no changes in the corresponding pooled SMD or in the significance of the results (Additional file 3: Figure S2), which indicated that our results were significantly robust to the study-selection process.

**Fig. 4:**
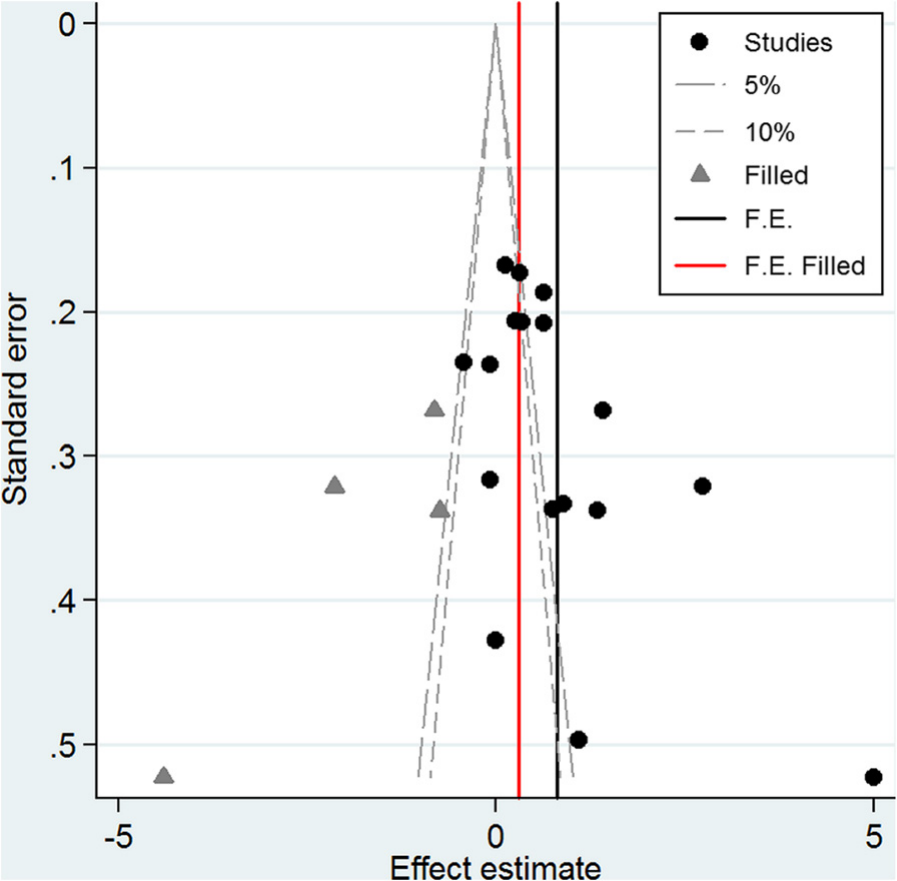
Contour-enhanced funnel plots for funnel plot asymmetry analysis of serum RBP4 level with GDM risk

Publication bias was assessed using Begg’s funnel plot and Egger’s test. The results of Begg’s funnel plot reveal the presence of significant asymmetry. These results are consistent with the modified Egger linear regression test and Begg’s test (*t* = 2.50, *p* = 0.025; *Z* = 2.27, *p* = 0.023). In order to analyze the potential causes to the funnel plot asymmetry, we treated the contour-enhanced funnel plots with the trim-and-fill method. This treatment resulted in 4 filled studies, which were all in the region of *p* < 0.05 (Fig. 4). Hence, the cause of the asymmetry may be more likely to be due to factors other than publication bias, such as variable study quality. After the “unpublished” (filled) studies are included, higher serum RBP4 level is still related to GDM risk (SMD = 0.300, 95 % CI [0.189, 0.411]).”

### Association between rs3758539 Polymorphisms and GDM Risk

**Fig. 5:**
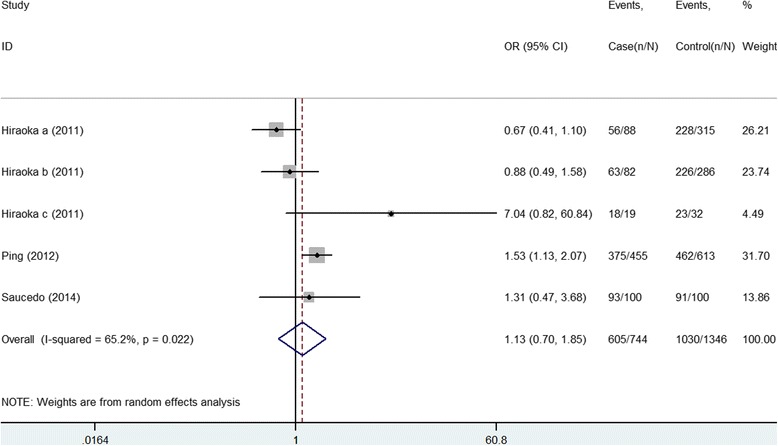
Meta-analysis for the association of rs3758539 polymorphism with GDM risk (GG vs. (GA + AA))

The meta-analysis concerning rs3758539 included three studies with a total of 1012 cases and 1605 controls. The dominant genetic model shows non-significant association between GG vs. GA + AA (OR = 1.133, 95 % CI [0.695, 1.846]). The following results were obtained only including two studies [8, 9]: G allele vs. A allele (OR = 1.093, 95 % CI [0.687, 1.739]), GG + GA vs. AA (OR = 1.123, 95 % CI [0.508, 2.483]), and GG vs. AA (OR = 1.124, 95 % CI [0.505, 2.502]) (Table 4, Fig. 5). Publication bias was not detected owing to the small number of available studies. During the sensitivity analysis, one eligible study was excluded each time to investigate the influence of the individual dataset on the pooled OR. The results show that the corresponding pooled OR and significant results did not change materially (Additional file 4: Figure S3), indicating that our results were significantly robust.

**Table 4:** Summary of different comparative results of rs3758539 polymorphism

Genetic model		No of case	No of control	OR (95 % CI)	Z	*p* value	I^2^%	P_het_	Effect model
Dominant	GG versus GA + AA	1012	1605	1.133 [0.695–1.846]	0.50	0.617	65.2	0.022	R
Recessive	GG + GA versus AA	893	1473	1.123 [0.508–2.483]	0.29	0.775	8.3	0.336	F
Additive	GG versus AA	893	1473	1.124 [0.505–2.502]	0.29	0.775	0.9	0.365	F
Allele	G versus A	912	1505	1.093 [0.687–1.739]	0.37	0.709	68.3	0.024	R

*P*_*het*_ = *p* value for heterogeneity, *OR* = adds ratio, *CI* = confidence interval, *F* = fixed-effect model, *R* = random-effect model

## Discussion

### Main findings

Our meta-analysis shows that higher prenatal serum RBP4 level is related to GDM risk, but this association cannot be shown in the first-trimester and postpartum periods. The GDM diagnostic criteria affected the strength of association between RBP4 level and GDM risk. Adopting a higher threshold of OGTT would result in a larger difference of serum RBP4 level between GDM women and controls. *RBP4* rs3758539 polymorphisms may not be associated with GDM risk.

### Interpretation

We pooled 17 studies (647 cases and 620 controls) in which the BMI-matched strategy was used, and then analyzed the relationship between serum RBP4 levels and GDM risk. Our results indicated that high serum RBP4 levels were related to GDM risk. We performed subgroup analyses of the effects of gestational age at blood sampling and diagnostic criteria. The studies included in the meta-analysis covered all periods during the pregnancy and only one study concerned the postpartum situation. The pooled results of second- and third-trimester subgroups were consistent with the overall result, GDM gravidas have higher serum RBP4 level than the controls. No statistically significant association was found between serum RBP4 levels and GDM risk in the postpartum subgroup and first-trimester subgroup. In the postpartum study blood samples were collected immediately after delivery. High exertion at delivery, which requires high output of energy, might temporarily enhance insulin sensitivity [16], and specific metabolic state at delivery might produce a difference between postpartum and prenatal results. The negative result of first-trimester subgroup reveals that with the insulin resistance level increasing during pregnancy, the magnitude of the effect of RBP4 may change. However, the inclusion of only a single study in postpartum subgroup and “before 24 weeks” subgroup could potentially make the stratified analysis unreliable. Therefore, the results must be interpreted with caution, and need to be validated using additional studies that include a large number of samples covering various periods during pregnancy and after delivery. In the subgroup analyses based on diagnostic criteria, both of the two subgroups show results consistent with the overall meta-analysis. And the pooled SMD of rigorous criteria subgroup is higher than the pooled SMD of ADA criteria subgroup. Although the result of ADA criteria subgroup had no statistical significance, we still can get further hints: higher blood glucose level at fasting and OGTT is associated with higher serum RBP4 levels. This finding was consistent with several published articles [17, 18, 21, 23, 31], which revealed that blood glucose and serum RBP4 levels presents a modest dose–response relationship. Thus, the results of subgroup analysis based on diagnostic criteria confirmed our hypothesis that RBP4 is related to glycometabolism in GDM.

Two articles reported the odds ratio about serum RBP4 level between women with and without GDM estimated from logistic regression models. The study by Abetew et al. [29] revealed that women in the highest quartile for serum RBP4 had a 1.89-fold higher risk of GDM compared with women in the lowest quartile (95 % CI: 1.05–3.43). However, this relationship did not reach statistical significance after adjustment for maternal race/ethnicity, family history of diabetes, and pre-pregnancy overweight status (adjusted OR: 1.54; 95 % CI: 0.82–2.90). In the study by Fruscalzo et al. [32], serum RBP4 level was not involved in the final multivariate logistic regression model after adjustment for maternal age, pre-pregnancy BMI, tobacco smoke, macro-region of origin, familial history of diabetes and so on.

The coding region of *RBP4* is located at chromosome 10q23–24 in humans, contains 5 exons and 6 introns, and has been linked to increased risk for type 2 diabetes in various populations [46, 47]. Numerous single nucleotide polymorphisms (SNPs), including +5,398 C > T, +8,201 T > A, +8,204 T > A, rs17484721, rs36035572, rs3758539, rs3758539, rs10882273, rs36014035, and rs34571439, have been identified as genetic markers for type 2 diabetes or insulin resistance [48–56]. Some of these SNPs are associated with circulating RBP4 level or RBP4 expression in visceral adipose tissues [48, 55, 57]. In this study, we investigated the relationship between the *RBP4* rs3758539 polymorphism and susceptibility to GDM, but did not find any significant association. Among the five races included in the meta-analysis, significant results were only observed in Han Chinese. However, given the low frequency of the minor allele of rs3758539 in these ethnic groups [11, 13] and the small OR [12], non-significant results could be attributed to the small sample size. Therefore, additional studies of a comparatively larger scale are required to further validate this result.

To the best of our knowledge, this is the first meta-analysis that has evaluated the relationship between genetic variants of *RBP4* and the risk of GDM. However, the study has certain limitations. First, the subgroup analysis performed based on gestational age at blood sampling included only one postpartum study, which makes the stratified analysis unreliable. Moreover, the meta-analysis of RBP4 polymorphisms involved a small number of samples and studies. Therefore, the results must be interpreted with caution. Second, funnel-plot asymmetry can confound the interpretation of meta-analyses. Here, the observed funnel-plot asymmetry might have resulted from poor methodological quality related to the small size and heterogeneity of the studies, in addition to publication bias [35, 44, 45]. Third, the sample size of each individual study of RBP4 levels and GDM is relatively small and all these studies are case–control studies. These factors affect the quality of individual studies included in the meta-analysis. Last, our study only included articles that featured English-language abstracts, and the main text written in English or Chinese, and this might have resulted in a language bias.

## Conclusions

The results of this meta-analysis support the hypothesis that RBP4 is a modest independent risk factor for GDM (i.e., nonobese patients with GDM might express RBP4 at abnormal levels). The serum RBP4 level is associated with the risk of GDM. However, the association in the first-trimester and postpartum period should be validated by further research. The association between *RBP4* rs3758539 polymorphism and GDM risk was not confirmed.

## Additional files

Additional file 1: Table S1. Adjusted covariates of all eligible studies for the association with serum RBP4 levels and GDM. (DOCX 18 kb)

Additional file 2: Figure S1. PRISMA flow diagram of study selection process (a) genetic variants and (b) serum concentration. (TIFF 4988 kb)

Additional file 3: Figure S2. The results of sensitivity analysis of serum RBP4 level with GDM risk. (TIFF 8567 kb)

Additional file 4: Figure S3. The results of sensitivity analysis of rs3758539 (GG vs. GA + AA) with GDM risk. (TIFF 5448 kb)

## Abbreviations

ADA: American Diabetes Association
BMI: body mass index
CI: confidence interval
GDM: gestational diabetes mellitus
HWE: Hardy–Weinberg equilibrium
NDDG: National Diabetes Data Group
OGTT: oral glucose tolerance test
OR: odds ratio
PEPCK: phosphoenolpyruvate carboxykinase
RBP4: retinol-binding protein-4
SD: standard deviation
SEM: standard error of mean
SMD: standardized mean difference
SNPs: single nucleotide polymorphisms
WHO: World Health Organization
