# Supplementary material for: Serum level and polymorphisms of retinol-binding protein-4 and risk for gestational diabetes mellitus: a meta-analysis
Source: BMC Pregnancy Childbirth. 2016 Mar 14;16:52. doi: 10.1186/s12884-016-0838-7 (PMC4791876; doi:10.1186/s12884-016-0838-7)
Supplement: Supplementary file 1 — Adjusted covariates of all eligible studies for the association with serum RBP4 levels and GDM. (DOCX 18 kb) [file 12884_2016_838_MOESM1_ESM.docx]

| Study | Year | Adjusted Covariates |
| --- | --- | --- |
| Chan a[16] | 2007 | Age, Gravidity, Parity, BMI at GCT, Gestational age at delivery, BMI at delivery, Fetal birth weight |
| Chan b[16] | 2007 | Age, Gravidity,Parity, BMI at GCT, Gestational age at delivery, BMI at delivery, Fetal birth weight |
| Kim[17] | 2008 | Age, Height, Weight at pre-pregnancy, Weight gain during pregnancy, Gestational age |
| Lewandowski*[19] | 2008 | Age, BMI before gestation, BMI at 28 weeks of gestation, sICAM-1, SVCAM-1 |
| Sun[20] | 2009 | Age, Gestational age at sampling, BMI at sampling |
| Klein a[21] | 2010 | Age, Parity, BMI at OGTT, Gestational age at OGTT, HbAlc at OGTT |
| Klein b[21] | 2010 | Age, Parity, BMI at OGTT, Gestational age at OGTT, HbAlc at OGTT |
| Su[23] | 2010 | Age, Pre-pregnancy BMI, BMI at GCT, Gestational age at GCT, Pre-pregnancy SBP/DBP,SBP/DBP at GCT, Triglycerides, Total cholesterol, HDL cholesterol, LDL cholesterol, Fasting insulin |
| Tepper[24] | 2010 | Age, Maternal BMI, Maternal weight gain, Infant birth weight, Gestational age at birth, Fasting insulin, Fasting leptin |
| Kuzmicki a*[26] | 2011 | Age, Parity, Gestational age, Pre-pregnancy BMI, Current BMI, Birth weight, HDL-cholesterol, LDL-cholesterol, Triglycerides, CRP |
| Kuzmicki b*[26] | 2011 | Age, Parity, Gestational age, Pre-pregnancy BMI, Current BMI, Birth weight, Total cholesterol, HDL-cholesterol, LDL-cholesterol, Triglycerides, CRP |
| Chen[25] | 2011 | Pre-pregnancy BMI , Weight gain during pregnancy, Gestational age,Gravidity, Parity, SBP, Birth weight, Total cholesterol, LDL-cholesterol |
| Ping[12] | 2012 | Age, Pre-pregnancy BMI, Gestational age at OGTT, WBC |
| Skvarca*[28] | 2012 | Age, Gestation age, BMI before pregnancy, BMI during pregnancy, Fasting insulin, Adiponectin, Leptin, Resistin, Visfatin |
| Liang[31] | 2014 | Age, Gestational week of sample collection, Pre-pregnant BMI, Glycosylated hemoglobin |
| Fruscalzo[32] | 2015 | Age, Gestational age at sampling, CRL, Pre-pregnancy BMI, Academic degree, Tobacco smoke, Mode of conception, Mode of delivery, Gestational age at delivery |
| Du[33] | 2015 | Age, Pre-pregnancy BMI, LDL, Total cholesterol , Gestational age at delivery, Maternal weight gain, Gravidity, Parity |

Supplementary Table 1. Adjusted covariates of all eligible studies for the association with serum RBP4 levels and GDM.

GCT = glucose challenge test; BMI = body mass index; sICAM-1, soluble intercellular adhesion molecule-1; sVCAM-1, soluble vascular cell adhesion molecule-1; OGTT, oral glucose tolerance test; HbA1c, hemoglobin A1c; SBP/DBP, systolic/diastolic blood pressure; HDL, high-density lipoprotein; LDL, low-density lipoprotein; CRP, high sensitive C-reactive protein; WBC, white blood cell; CRL, crown-rump length.
