# Supplementary figures and images for: Serum level and polymorphisms of retinol-binding protein-4 and risk for gestational diabetes mellitus: a meta-analysis
Source: BMC Pregnancy Childbirth. 2016 Mar 14;16:52. doi: 10.1186/s12884-016-0838-7 (PMC4791876; doi:10.1186/s12884-016-0838-7)

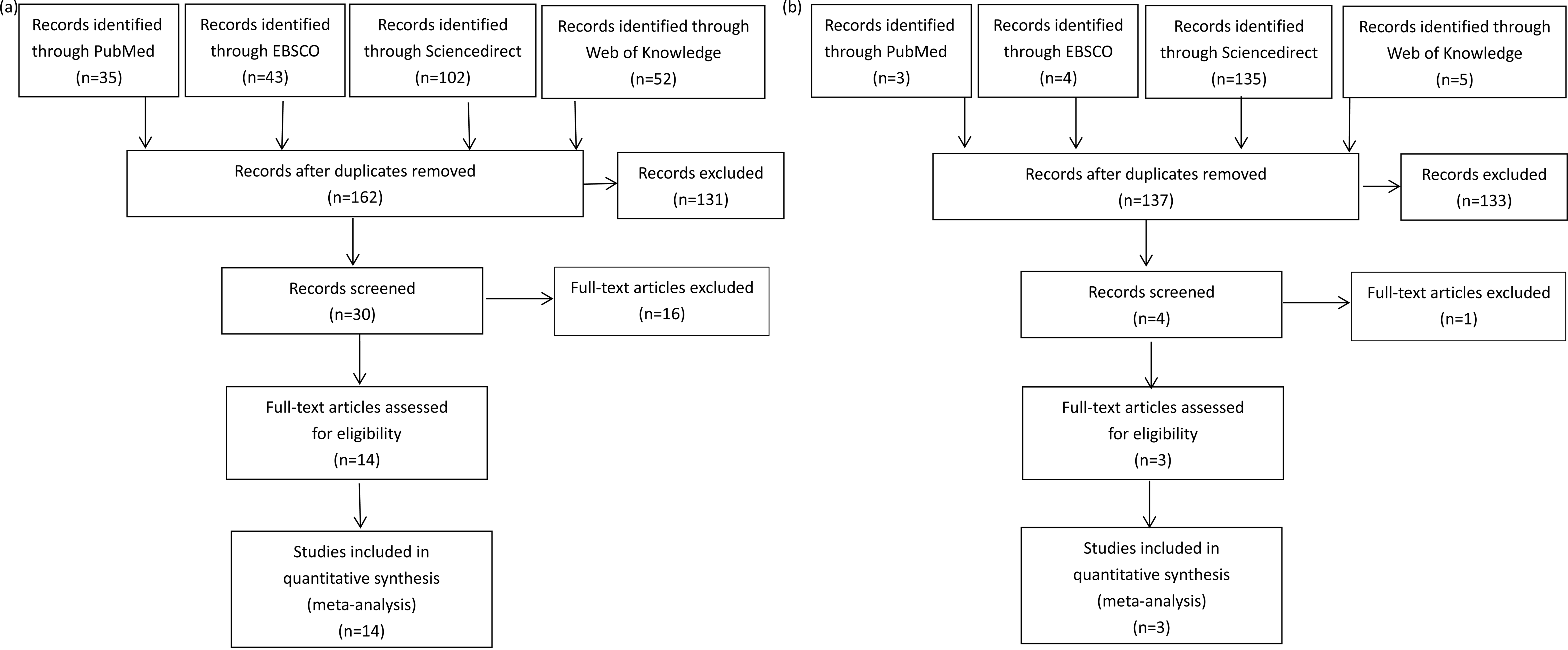

Supplement: Supplementary file 2 — PRISMA flow diagram of study selection process (a) genetic variants and (b) serum concentration. (TIFF 4988 kb) [file 12884_2016_838_MOESM2_ESM.tiff]

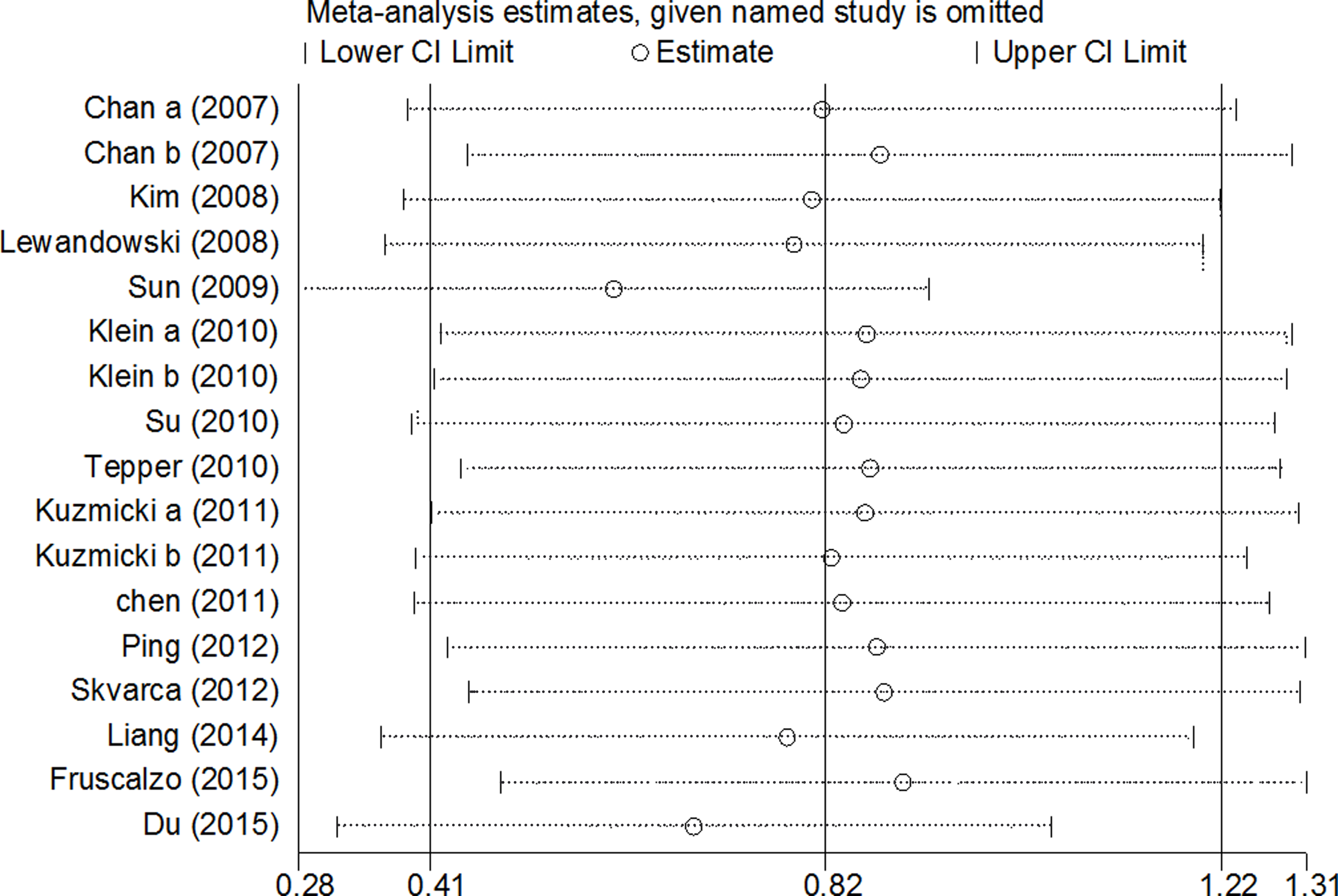

Supplement: Supplementary file 3 — The results of sensitivity analysis of serum RBP4 level with GDM risk. (TIFF 8567 kb) [file 12884_2016_838_MOESM3_ESM.tiff]

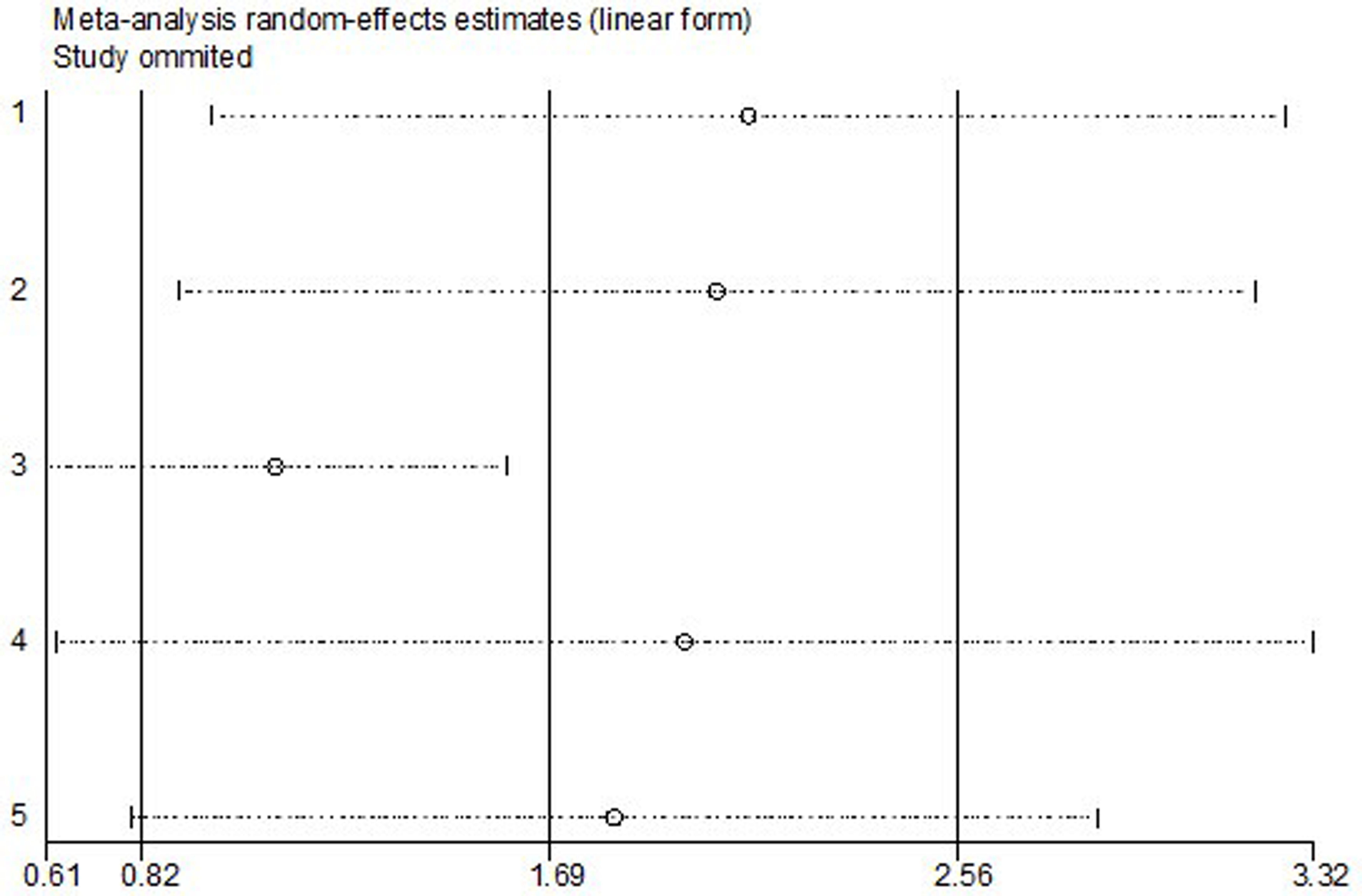

Supplement: Supplementary file 4 — The results of sensitivity analysis of rs3758539 (GG vs. GA + AA) with GDM risk. (TIFF 5448 kb) [file 12884_2016_838_MOESM4_ESM.tiff]
